# Unraveling the link between PTBP1 and severe asthma through machine learning and association rule mining method

**DOI:** 10.1038/s41598-023-42581-5

**Published:** 2023-09-16

**Authors:** Saeed Pirmoradi, Seyed Mahdi Hosseiniyan Khatibi, Sepideh Zununi Vahed, Hamed Homaei Rad, Amir Mahdi Khamaneh, Zahra Akbarpour, Ensiyeh Seyedrezazadeh, Mohammad Teshnehlab, Kenneth R. Chapman, Khalil Ansarin

**Affiliations:** 1 Clinical Research Development Unit of Tabriz Valiasr Hospital, Tabriz University of Medical Sciences, Tabriz, Iran; 2 Kidney Research Center, Tabriz University of Medical Sciences, Tabriz, Iran; 3 Rahat Breath and Sleep Research Center, Tabriz University of Medical Science, Tabriz, Iran; 4 Faculty of Advanced Medical Sciences, Tabriz University of Medical Sciences, Tabriz, Iran; 5 Tuberculosis and Lung Disease Research Center, Tabriz University of Medical Sciences, Tabriz, Iran; 6 Department of Electric and Computer Engineering, K.N. Toosi University of Technology, Tehran, Iran; 7 Division of Respiratory Medicine, Department of Medicine, University of Toronto, Toronto, ON Canada

**Keywords:** Respiratory tract diseases, Machine learning, Gene expression

## Abstract

Severe asthma is a chronic inflammatory airway disease with great therapeutic challenges. Understanding the genetic and molecular mechanisms of severe asthma may help identify therapeutic strategies for this complex condition. RNA expression data were analyzed using a combination of artificial intelligence methods to identify novel genes related to severe asthma. Through the ANOVA feature selection approach, 100 candidate genes were selected among 54,715 mRNAs in blood samples of patients with severe asthmatic and healthy groups. A deep learning model was used to validate the significance of the candidate genes. The accuracy, F1-score, AUC-ROC, and precision of the 100 genes were 83%, 0.86, 0.89, and 0.9, respectively. To discover hidden associations among selected genes, association rule mining was applied. The top 20 genes including the PTBP1, RAB11FIP3, APH1A, and MYD88 were recognized as the most frequent items among severe asthma association rules. The PTBP1 was found to be the most frequent gene associated with severe asthma among those 20 genes. PTBP1 was the gene most frequently associated with severe asthma among candidate genes. Identification of master genes involved in the initiation and development of asthma can offer novel targets for its diagnosis, prognosis, and targeted-signaling therapy.

## Introduction

Asthma is a common chronic airway disease and a global public health problem, affecting nearly 300 million people around the world^1^ and ranked 16th among the main causes of years of life loss with a disability^2^. Two recognized asthma endotypes exist based on the absence or presence of type 2 (T2) airway inflammation. Its most common form, Type 2 asthma is an eosinophilic chronic airway inflammation characterized by recurrent and reversible airway narrowing and obstruction, airway hyper-responsiveness, mucous hypersecretion, and oftentimes airway wall remodeling^3^. These effects are associated with T-helper2 (Th2) cells, innate lymphoid cells, eosinophils, mast cells, and B cells all interconnected by a complex interplay of chemokines and cytokines. Although our understanding of asthma is far from complete, there is a growing understanding of the molecular mechanisms that underlie common clinical phenotypes^4^.

Despite the worldwide distribution of asthma guidelines and advances in the treatment of asthma, a significant number of patients experience poor control of asthma stated as difficult-to-treat or severe asthma. Severe asthma comprises 3–10% of the asthmatic adult population^5,6^ but accounts for more than 60% of the expenses^7,8^. The management of this type of asthma has many challenges related to adherence, psychosocial morbidity, and treatment. Patients with severe asthma need repeated oral corticosteroid therapy that frequently is connected with a variety of adverse events^9^. Current therapies are insufficient for patients with severe asthma^10–12^. Moreover, currently, there is no marker to define the length of therapy or assess the response. Therefore, the development of novel targeted therapies and biomarkers can introduce precision medicine for these patient and this form of asthma calls for more detailed studies to target the exploration of the pathologic mechanisms at genomic and molecular levels. Exploring severe asthma mechanisms at the genomic level may shed light on disease processes helping to pave the way for a better understanding of the process with potential therapeutic consequences. Recent studies have focused on the immune cells^13,14^ and signaling pathways in the asthma process^15^, generating a variety of associated gene candidates of interest.

In the U-BIOPRED (Unbiased Biomarkers in Prediction of Respiratory Disease Outcome) dataset^16^, researchers studied severe asthma based on omics data obtained from different tissue/cells including bronchial biopsies, bronchial and nasal brushings, sputum, urine, and blood. The current study aimed to employ and analyze the gene expression data provided in the U-BIOPRED study. In the present study, we focused on gene expression in blood samples, since inflammatory and immune cells, as well as systemic treatments, are transported through the blood to reach the lungs^17^; providing actual insights into the complex gene interactions associated with asthma severity.

New technologies are changing medicine, and this revolution starts with health data, including clinical images, genomic, and prescribed therapy data^18^. We are witnessing the exponential growth of machine learning applications in health-related information besides the traditional analysis techniques, which are not suitable for managing this vast amount of data^19^. Furthermore, applying state-of-the-art machine learning-based algorithms helped us to clarify and get a better understanding of trigger genes in severe asthma, yielding the potential to comprehensively characterize the actual mechanism of severe asthma at the gene level by considering gene expression and gene–gene interactions.

## Materials and methods

### Study population

Asthma-related gene expression datasets were obtained from the Gene Expression Omnibus (GEO) repository of the National Center for Biotechnology Information (NCBI). In this study, three datasets were employed, two of which are known as GSE69683^17^ (https://www.ncbi.nlm.nih.gov/geo/query/acc.cgi?acc=GSE69683) and GSE76262^20^ (https://www.ncbi.nlm.nih.gov/geo/query/acc.cgi?acc=GSE76262). Table 1 elaborates more on the represented classes of these two datasets. In GSE69683 and GSE76262, gene expression levels were extracted from blood and sputum, respectively. Two datasets were measured based on the same platform, referred to as GPL13158. This platform applied the Affymetrix Human Genome HT U133 + PM array technique, in which gene expression levels are reported using 54,715 Probes for 20,277 genes. These datasets are subsets of Unbiased BIOmarkers in PREDiction of respiratory disease outcomes (U-BIOPRED), a multi-center prospective cohort study in which the researchers collected gene expression data from 16 clinical centers in 11 European countries.

**Table 1:** Datasets information.

GSE69683				GSE76262		
Male: 223, Female: 275				Male: 63, Female: 76		
Age: N/A				Age = 49 ± 14.29		

Asthma status				Asthma status		
Severe		Moderate	Non-asthmatic	Severe	Moderate	Non-asthmatic
Non-smoking	Smoking	Non-smoking	Non-smoking			
246	88	77	87	93	25	21

Another asthma-related dataset is GSE110551^21^ (https://www.ncbi.nlm.nih.gov/geo/query/acc.cgi?acc=GSE110551) containing gene expression levels of 78 patients with asthma and 78 individuals unaffected by asthma. This dataset is available on the NCBI-GEO website, and gene expression level measurement was carried out by using the GPL 570 platform. This platform utilizes the Affymetrix Human Genome U133 Plus 2.0 Array technique, to which 54,675 probes were applied to record gene expression data. In GSE110551, a variety of important clinical information such as Body Mass Index (BMI) is reported in addition to asthma status. BMI is a significant factor in determining the obesity status of donors and this property gives GSE110551 a unique position to facilitate the study of the relationship between asthma and obesity at the gene level. Table 2 represents the sample counts of this database in each category. The datasets were used during the current study are available in the GEO repository (https://www.ncbi.nlm.nih.gov/geo/). This study was conducted according to the principles of the Declaration of Helsinki (2013) and informed consent was obtained from all subjects and/or their legal guardian(s).

**Table 2:** GSE110551 dataset details.

GSE110551			
Male: 59, Female: 97		Age: 41.76 ± 12.41	

Severe asthma		Non-asthma	
Obese	Non-obese	Obese	Non-obese
39	39	39	39

### Methodology

**Figure 1:**
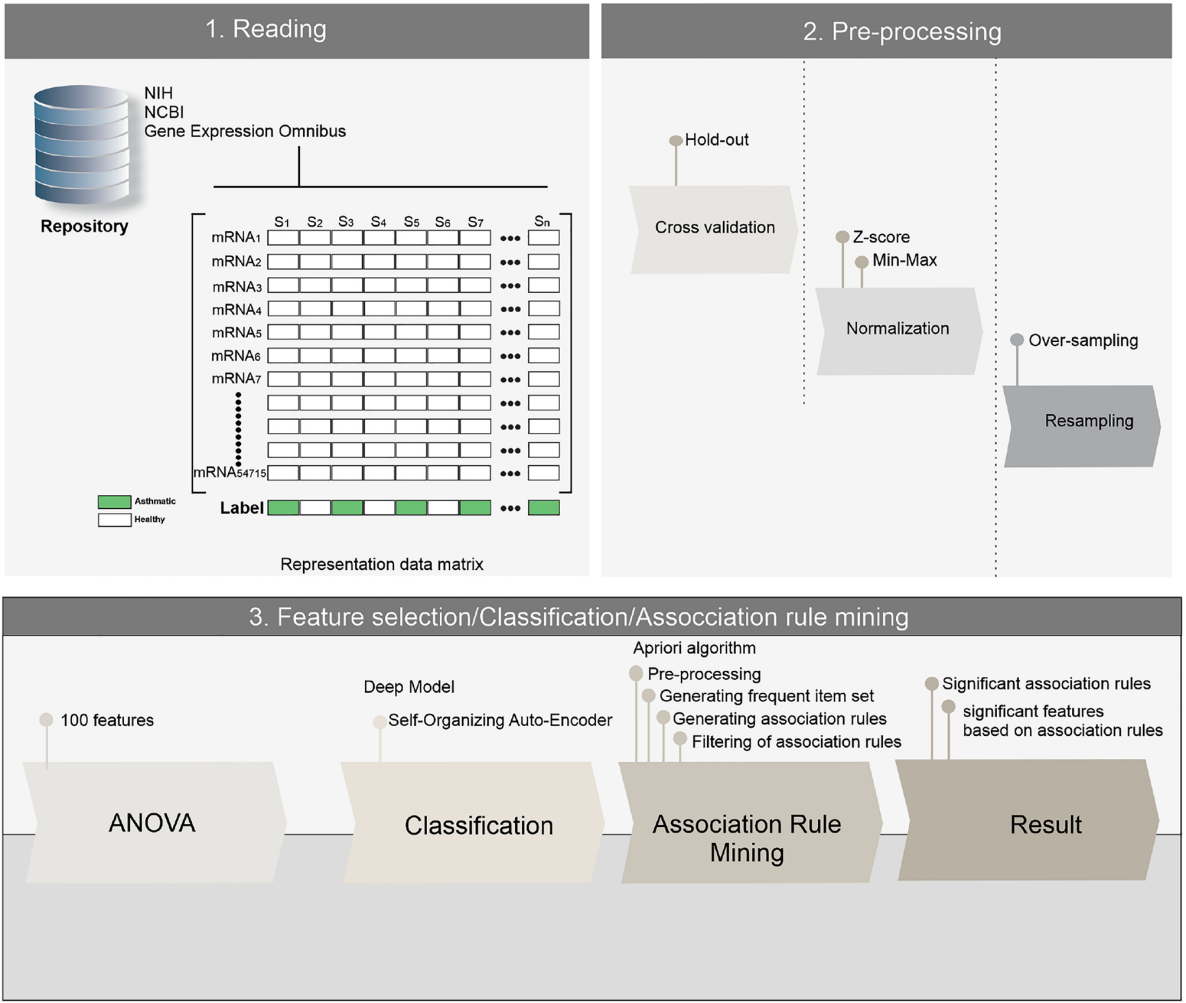
The overview of the proposed method. Five main steps, including reading, preprocessing, feature selection, classification, and association rule mining were performed on mRNA transcript data. (1) Each dataset was downloaded from the NCBI-GEO repository in the reading step. Clinical and gene expression data were extracted for all samples. (2) Three sub-steps including cross-validation, normalization, and resampling were used in the preprocessing step. (3). The F-value for 54,715 features was calculated by Feature selection via the ANOVA method, where the high amount of F-value shows genes with more relevance to severe asthma. In order to assess the differentiation power of selected genes, a classifier model was used. At the first level, the Association Rule Mining technique discovered the hidden relationship between selected genes and severe asthma, and at the second level, it identified the complex relationship among selected genes.

The proposed method in this study has five main steps; Reading, Pre-processing, Feature Selection, Classification, and Association Rule Mining (Fig. 1).

#### Reading step

In the reading stage, each dataset was downloaded from the NCBI-GEO repository. Subsequently, clinical data and gene expression were extracted for all samples. Each sample had a features vector (with 54,715 rows) and a corresponding label. The class label had two values, 0 for the case (severe asthma) and 1 for the control (non-asthma) samples. Tables 2 and 3 represent case–control details for GSE69683, GSE76262, and GSE110551 datasets.

**Table 3:** Case–control details for GSE69683 and GSE76262.

GSE69683		GSE76262	
Case (severe asthma)	Control (Non-asthmatic)	Case (severe asthma)	Control (Non-asthmatic)
334	87	93	21

#### Pre-processing step

The pre-processing step included three sub-steps; cross-validation, normalization, and resampling. Data were split into two parts using the hold-out cross-validation method, at a 70% to 30% ratio, for training and test portions, respectively. Only the training data were used during the pre-processing, feature selection, model training for classification, and association rule mining. On the other hand, test data were utilized only within the performance evaluation of feature selection and classification steps.

For the normalization of the training data, two approaches were applied during (i) feature selection and (ii) classification; Min–max for the former and Z-score for the latter as described previously^22^. In order to address the side-effects of imbalanced data, we applied random duplicate oversampling technique to bring parity between minority and majority classes, only for the training part of the data of course.

#### Feature selection step

Feature selection (FS) is an essential step for feature processing in machine learning, pattern recognition, and data mining. Although FS is rooted deeply in the statistics literature, it plays an important role in machine learning applications. FS algorithms are applied to input data to eliminate irrelevant and redundant features, especially in high-dimensional spaces such as genomics data. By utilizing FS methods, generally, the main goal is to assist the machine learning algorithm to focus on those aspects of the data that are more valuable for analysis and future predictions.

Applying high-throughput molecular analysis methods has given rise to more genomic datasets with higher dimensionality and more complexity. As the data dimensionality grows, it also becomes harder for biology researchers to interpret genomic interactions. Selecting a subset of relevant features using FS techniques not only facilitates the interpretation of genomics data by nominating biomarkers associated with diseases but can also improve classification performance by avoiding the curse of dimensionality that comes with high-dimensional spaces.

FS methods can be classified into four categories, consisting of filter, wrapper, embedded, and hybrid approaches^23^. Generally, filter methods use some scoring functions to rank features based on their differentiation strength. Filter methods detect the importance of features individually and do not consider possible interactions between them. In addition, they have a low computational cost due to not utilizing classifiers in the feature selection process; as a matter of fact, they are classifier-independent^24^.

Regardless of the major developments in the feature selection field, the analysis of high dimension- low sample size (HDLSS) data is an active area for research^25^. In the biology domain, genomics data fall into the HDLSS category and it is advisable to consider HDLSS methods for feature selection.

During this study, a serial combination method containing the analysis of variance (ANOVA) and association rule mining^22^ is used to select features in gene expression data as illustrated in Fig. 1. Later on, a classifier model is utilized to pinpoint the quality of selected features obtained after the ANOVA step. The classifier model would verify that selected features can exhibit highly differentiating characteristics; in order to discover possible hidden gene relationships, association rule mining is put to work subsequently.

### ANOVA

ANOVA is a simple and powerful method to compare the mean value of multiple groups (classes) in a dataset. It highlights any significant difference between the mean values of groups^26^. This method has many advantages including: being robust in point view violations of its assumptions, being more intuitive to analyze the interaction of two features, being effective even in datasets with imbalanced number of samples in target classes, and also being easy to generalize to more than two groups without increasing the Type 1 error. ANOVA is called F-statistic in statistics literature, and is calculated by Eq. (1)^27^.

1 $${F}_{value}= \frac{BMS}{WMS}$$

In Eq. (1), BMS and WMS are between mean squares and within mean squares, respectively. BMS and WMS are calculated by Eqs. (2) and (3).

2 $$BMS= \frac{BSS}{{df}_{B}}= \frac{\sum_{i=1}^{C}{n}_{i}{({\overline{x} }_{i}-\overline{x })}^{2}}{{df}_{B}}$$

3 $$WMS= \frac{WSS}{{df}_{W}}= \frac{\sum_{i=1}^{C}({n}_{i}-1){{\sigma }_{i}}^{2}}{{df}_{W}}$$

BSS and WSS are between sum squares and within sum squares, respectively. Where $\overline{x }$ = mean value of total samples, ${\overline{x} }_{i}$ = mean value of ith class, ${\sigma }_{i}$ = standard deviation of ith class, ${n}_{i}$ = sample number of ith class. ${df}_{B}$ = C − 1 and ${df}_{W}$ = N–C represent degree of freedom, in which N = number of total sample and C = Number of classes.

ANOVA assigns calculated F-values for all features and performs the ranking process accordingly. Features with high F-values are significant, since they represent better differentiation capabilities between classes.

#### Classification step

The classification process plays a major role in machine learning tasks. During this stage, the classifier model learns to separate classes of data based on the input features. This separation is performed using linear or non-linear boundaries and the performance of the classifier demonstrates how much the classes are separable.

The classifier model acts as a predictor of disease status, such as case–control, patient-healthy, cancer subtypes, etc., in medical applications. It can also indicate the quality of selected features if a feature selection step is performed previously. A classifier model with a decent performance verifies that selected features embody important differentiating characteristics to determine disease status, and the elimination of irrelevant features does not hurt the model’s differentiation ability.

We have employed a deep model for classification purposes and the proposed deep learning algorithm utilizes Self-Organizing Auto-Encoder (SOAE)^28^ as the building blocks of the deep model. The distinctive property of a self-organizing deep auto-encoder is that it can automatically determine its structure (number of layers and neurons) based on the input data.

#### Association rule mining step

Genes and their products (i.e., proteins and RNAs) in the human body act according to their exclusive functions, in a complicated and orchestrated way. Most of the classical methods in molecular biology fall short of providing an overall picture of gene functions and interactions. Nowadays, the DNA microarray technique is widely used to measure thousands of gene expression levels at a given time, under given conditions for any cells or tissues. Unlike traditional methods in molecular biology, the analysis of microarray data is challenging and not straightforward. The microarray data is reported in a matrix form of N × M, in which N rows and M columns represent genes (commonly thousands) and samples (generally hundreds), respectively. Biology researchers apply some computational strategies, such as clustering and bi-clustering, to analyze microarray data^29,30^. However, discovering the existing interactions between genes is not achievable using the same course of action, since most of the genes have functions in more than one gene network^31^. The Association Rule Mining (ARM) method is an innovative Gene Association Analysis (GAA) practice that can help discover such relationships.

Association rule mining is a useful methodology in the data mining area to uncover possible hidden connections in large and high dimensional, yet sparse data. Apriori is probably the most used association rule mining algorithm to date^32^; however, several improved algorithms are proposed as well, such as the Apriori-Hybrid algorithm^33^, fuzzy association rule algorithm^34^ and FP-Growth algorithm^35,36^.

We have employed the Apriori algorithm to generate frequent item sets and association rules for this study. The algorithm contains three main steps; generating frequent itemsets, generating association rules, and filtering, as shown in Fig. 1.

Selected rules and features in the association rule mining stage are evaluated using prior biological knowledge; either available in the literature or open-access biological databases. Furthermore, related association rules are studied to unmask the selected gene interactions. The main purpose of this step is to verify the biological significance of selected genes and their association rules.

### Ethical approval

This study was approved by the Ethics Committee of the National Institute for Medical Research Development (NIMAD), Teran, Iran (Ethical code: IR.NIMAD.REC.1398.099).

## Results

**Figure 2:**
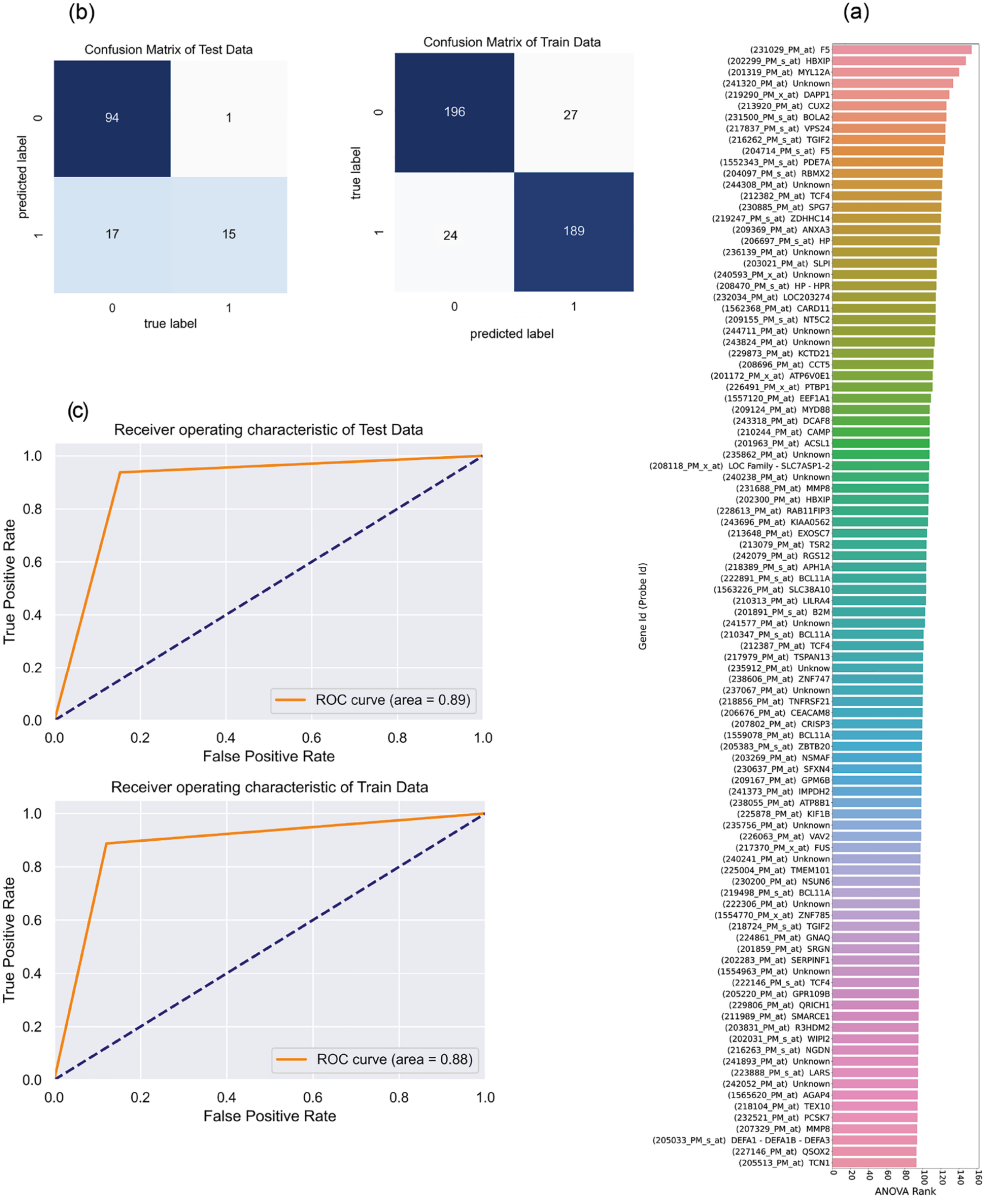
The results of feature selection and classification steps in the GSE69683 dataset. (**a**) Bar plot of F-value of top 100 probe ideas/gene symbols, calculated by ANOVA method. (**b**) Confusion matrix of training and test data, in which 0 and 1 were pointed to asthmatic and healthy groups, respectively. (**c**) ROC curve of training and test data. AUC-ROC is reported for each curve. *AUC-ROC* Area under the Curve of Receiver Operating Characteristic.

We propose a multi-step procedure to discover the gene(s) that control the disease process, by considering gene-asthma and gene–gene interactions in severe asthma (Fig. 1). In the wake of going through the reading and preprocessing steps, training data gets ready for the feature selection stage. With 54,715 features reported for 436 samples (case and control) in training data, we calculated the F-value using the ANOVA method. Features with higher F-values imply more relevance to severe asthma and help distinguish healthy and asthmatic cases more accurately. The top 100 genes (features) with the highest F-value (shown in Fig. 2a.) were selected as the leading features for the proceeding steps. We chose the “100” feature count threshold based on our experiments, ensuring that they embody good differentiation power while not sacrificing much of the performance.

We used a classifier model to evaluate the differentiation quality of selected genes. A deep neural network model based on SOAE (self-organizing auto-encoder) as a building block was utilized to classify new training data with 100 selected features. The accuracy, F1-measure, and AUC-ROC are applied to evaluate the classifier performance, Eqs. (4), (5), (6) and (7). [True Positive (TP), True Negative (TN), False Positive (FP), and False Negative (FN)]

4 $$Accuracy= \frac{TP+TN}{TP+FN+FP+TN}\times 100$$

5 $$F-measure= \frac{Precision\times Recall}{Precision+Recall},$$

where

6 $$Recall=\frac{TP}{TP+FN}$$

7 $$Precision=\frac{TP}{TP+FP}$$

Accuracy, F1-score, Precision, Recall, and AUC-ROC were reported for training and test data of GSE69683 in Table 4. The classification performance of training data (accuracy = 89% and AUC-ROC = 0.88) and test data (accuracy = 83% and AUC-ROC = 0.89) showed that the deep model was able to learn and classify severe asthmatic and healthy groups, achieving very good scores. The confusion matrix and ROC curve for both training and test data are displayed in Fig. 2b and c, respectively.

**Table 4:** The performance metrics for the classification step (SOAE Model).

	GSE69683									
	Train data					Test data				
	Precision	Recall	F_1_-score	Accuracy	AUC-ROC	Precision	Recall	F_1_-score	Accuracy	AUC-ROC
Asthmatic	0.91	0.88	0.89	0.89	0.88	0.97	0.84	0.90	0.83	0.89
Healthy	0.88	0.91	0.89			0.42	0.81	0.55		
Macro Avg	0.89	0.89	0.89			0.69	0.83	0.73		
Weighted Avg	0.90	0.89	0.89			0.90	0.83	0.86		

Following the classification step, we employed the association rule mining method to first, discover the possible hidden connections between nominated genes and severe asthma disease and second, figure out the complex relationships among selected genes. We applied a binning preprocess to gene expression values (or features). Each gene expression value can be categorized into one of three bins, namely low, intermediate, and high. This allowed us to convert continuous gene expression values to discrete gene expression itemsets. Moving on, the Apriori algorithm was used to generate frequent itemsets, and rules with minimum support and lift threshold values set to 0.28 and 1.1, respectively. All of the mentioned configurations resulted in the generation of 30,261,700 association rules, based on 387,848 frequent itemsets obtained through the Apriori algorithm.

**Figure 3:**
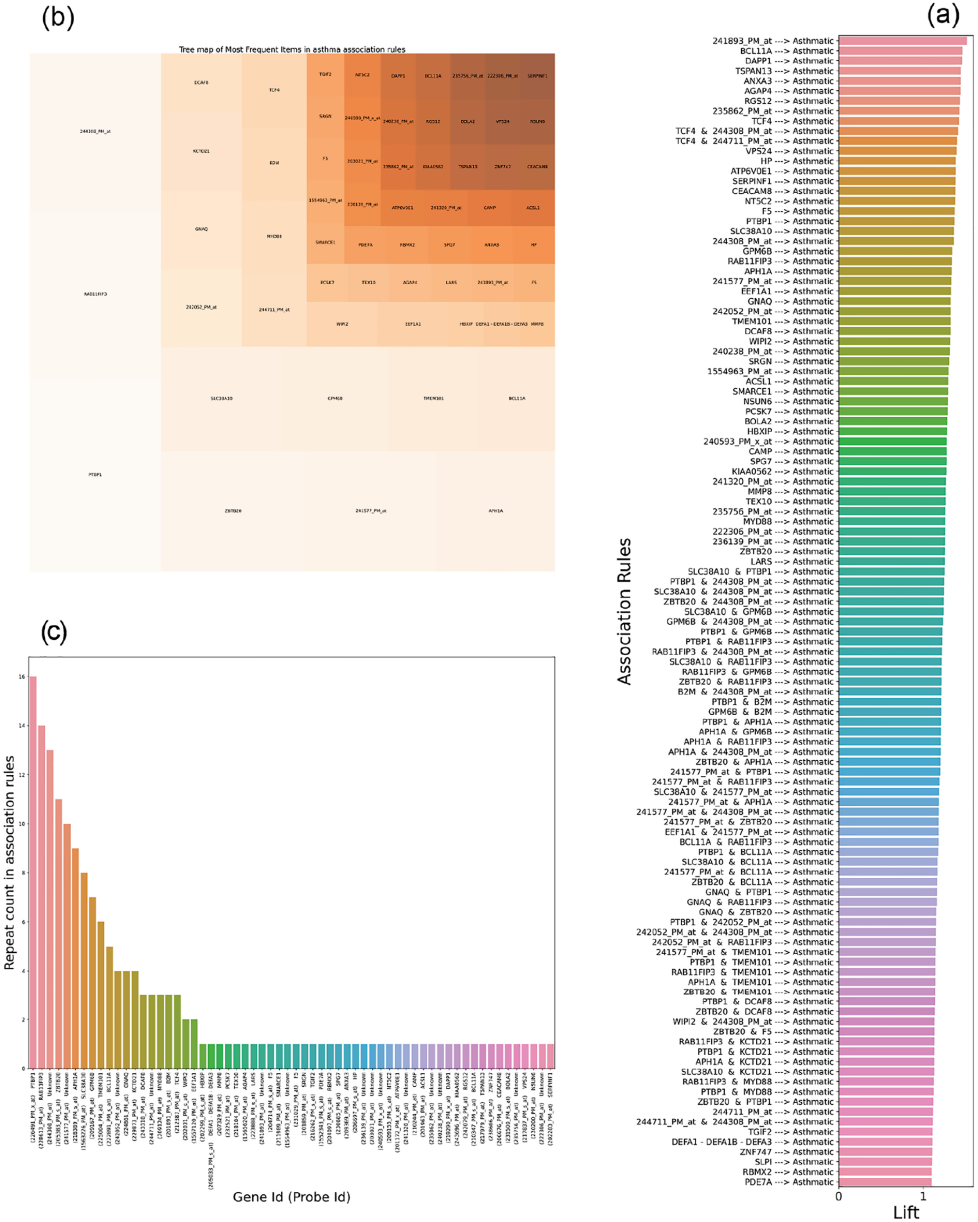
The performance of the association rule mining step in the GSE69683 dataset. (**a**) Bar plot of lift values of asthmatic association rules, generated by Apriori algorithm with minimum support 0.28 and minimum lift 1.1. (**b**) Tree map of most frequent items in asthmatic association rules, in which the most frequent probe/gene takes a square with the greatest space. (**c**) Bar plot of probe/gene repeat count in asthma association rules.

We selected 115 association rules with the consequent part relevant to severe asthma (Fig. 3a). Overall, 62 unique genes or probes could be identified from these rules, with varying appearance frequency which is shown in Fig. 3b and c. The PTBP1 (repeat count = 16), RAB11FIP3 (repeat count = 14), ZBTB20 (repeat count = 11), APH1A (repeat count = 9), SLC38A10 (repeat count = 8), GPM6B (repeat count = 7), TMEM101 (repeat count = 6), BCL11A (repeat count = 5), GNAQ (repeat count = 4), KCTD21 (repeat count = 4), TCF4 (repeat count = 3), B2M (repeat count = 3), DCAF8 (repeat count = 3), MYD88 (repeat count = 3), WIP12 (repeat count = 2), and EEF1A1 (repeat count = 2) are genes (or items) with the highest frequency of appearance amongst extracted rules. In addition, there are 4 frequent probes including 244308_PM_at (repeat count = 13), 241577_PM_at (repeat count = 10), 242052_PM_at (repeat count = 4), and 244711_PM_at (repeat count = 3); since gene symbol is not reported for these probes in Affymetrix guide, we refer to them as unknown genes with some numerical identifiers.

**Figure 4:**
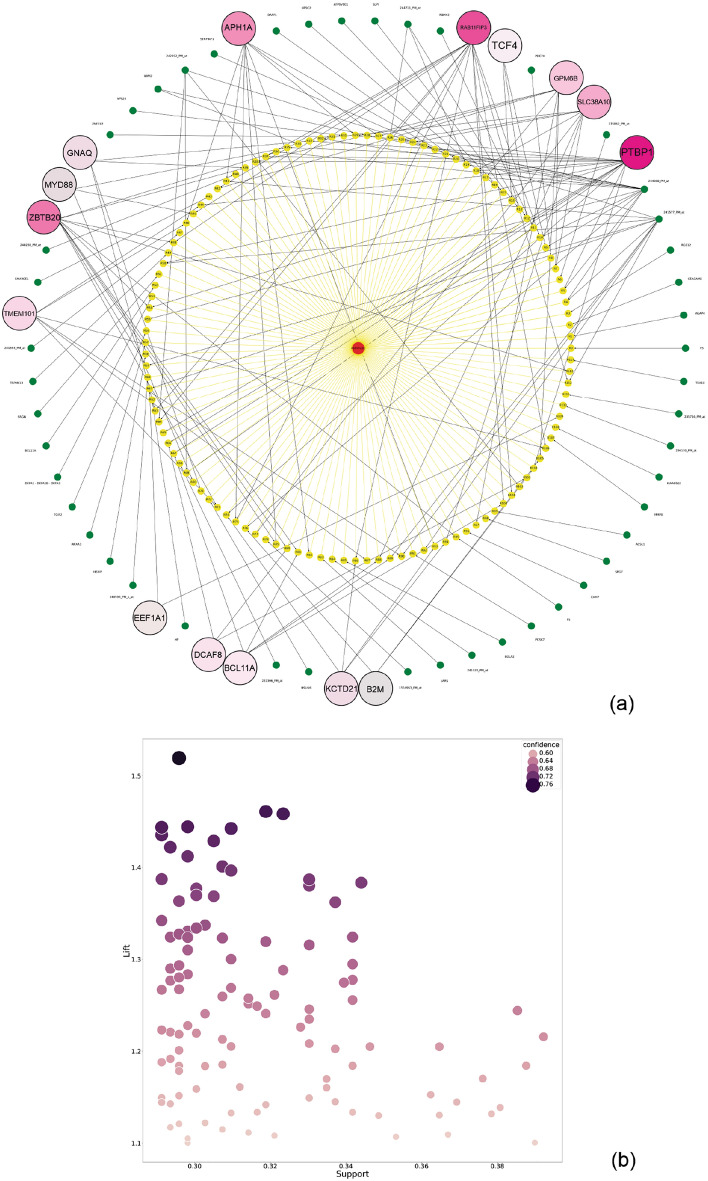
The relationship and specification of the asthma association rules in the GSE69683 dataset. (**a**) Graph network of severe asthmatic association rules, in which asthma and rules are presented by red and yellow colors, respectively. The most frequent probes/genes are displayed with the color spectrum based on repeat counts. (**b**) Strength distribution of severe asthmatic association rules according to their support, lift, and confidence.

Moreover, a graph network for severe asthma association rules and the strength distribution of severe asthma association rules according to their support, lift, and confidence values are shown in Fig. 4a and b. We presented example 'if–then' association rules in Table 5, where the antecedent contained PTBP1 (most frequent itemset with 16 repeat counts) for asthmatic condition.

**Table 5:** Asthmatic association rules, where the antecedent contained PTBP1.

	Antecedent			Consequent
If	PTBP1	ZBTB20	Then	Asthmatic
If	PTBP1	DCAF8	Then	Asthmatic
If	PTBP1	244308_PM_at	Then	Asthmatic
If	PTBP1	KCTD21	Then	Asthmatic
If	PTBP1	MYD88	Then	Asthmatic
If	PTBP1	RAB11FIP3	Then	Asthmatic
If	PTBP1	APH1A	Then	Asthmatic
If	PTBP1	BCL11A	Then	Asthmatic
If	PTBP1	B2M	Then	Asthmatic
If	PTBP1	241577_PM_at	Then	Asthmatic
If	PTBP1	SLC38A10	Then	Asthmatic
If	PTBP1	GPM6B	Then	Asthmatic
If	PTBP1	ZBTB20	Then	Asthmatic
If	PTBP1	242052_PM_at	Then	Asthmatic
If	PTBP1	GNAQ	Then	Asthmatic
If	PTBP1	TMEM101	Then	Asthmatic

**Figure 5:**
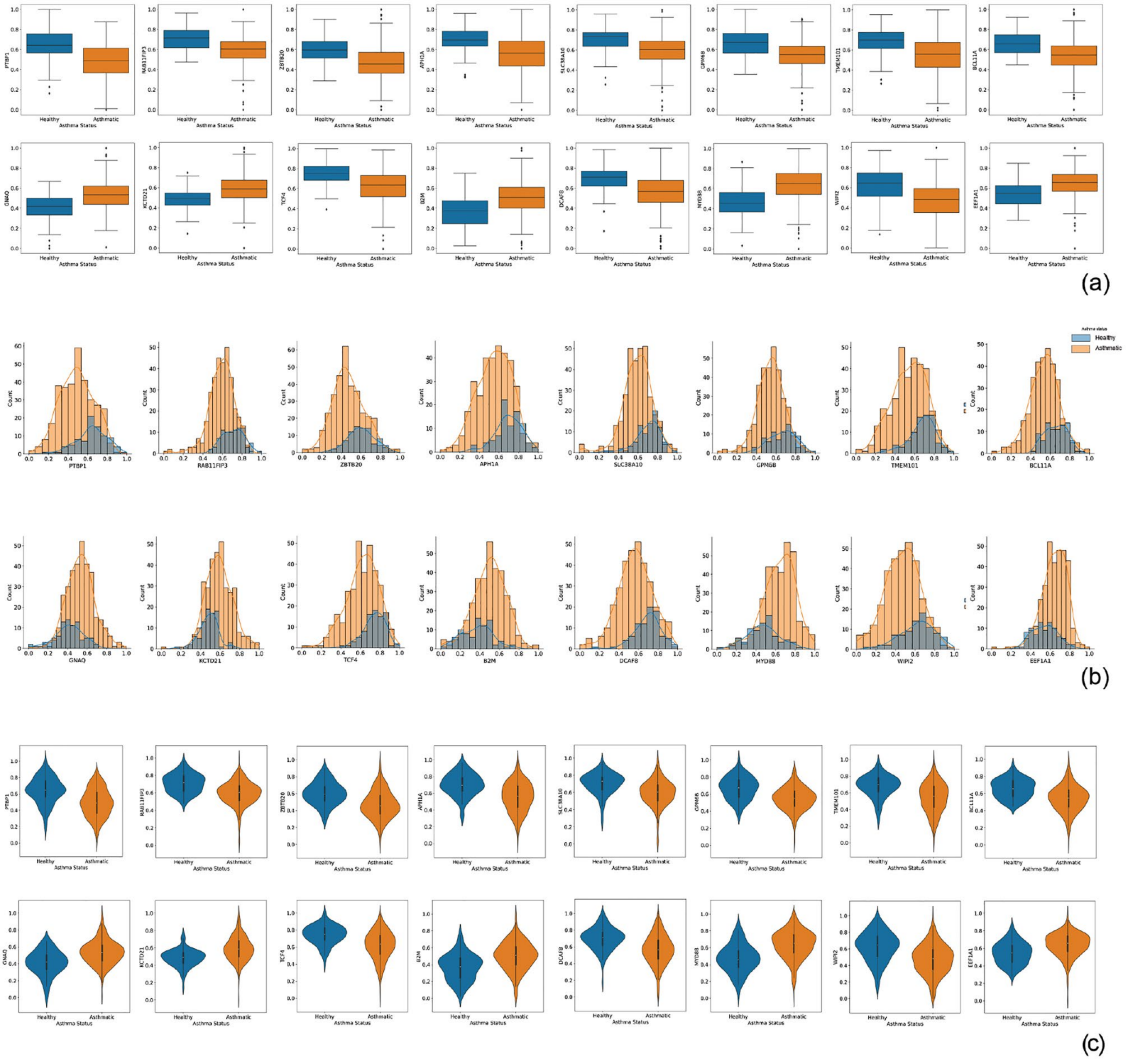
The specification of the selected genes is based on the association rule mining step. (**a**) Box plot of selected genes for severe asthmatic and healthy groups, expression values of genes have been normalized in the range of 0 to 1 by the min–max method. (**b**) Probability Density Function plot of selected genes for severe asthmatic and healthy groups. (**c**) Violin plot of selected genes for severe asthmatic and healthy groups.

Comparing the Boxplots of asthma and healthy groups, it is obvious that there is a significant difference in all reported genes (Fig. 5a). Density distribution and violin plots are also available for reported genes in Fig. 5b and c.

**Figure 6:**
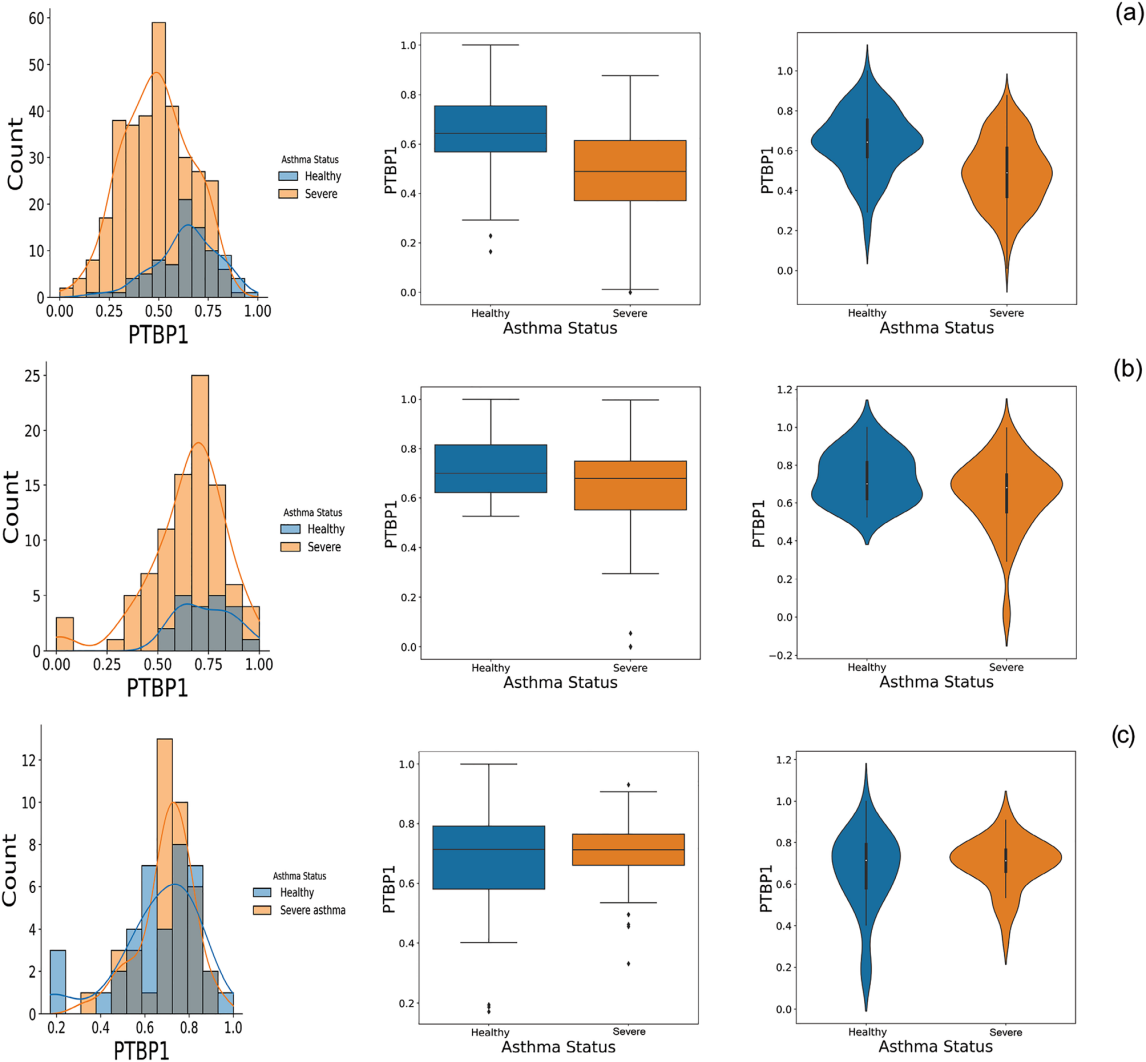
The comparison of the PTBP1 expression in GSE69683, GSE76262, and GSE110551 datasets. (**a**) Probability Density Function plot, Box plot, and Violin plot of PTBP1 expression for severe asthmatic and healthy groups in GSE69683. (**b**) Probability Density Function plot, Box plot, and Violin plot of PTBP1 expression for severe asthmatic and healthy groups in GSE76262. (**c**) Probability Density Function plot, Box plot, and Violin plot of PTBP1 expression for severe asthmatic and healthy groups in GSE110551.

The PTBP1 was the most frequent itemset with 16 repeat counts in asthmatic association rules; it also appears to be impactful in steering moderate asthma to severe asthma. As a result, we decided to investigate this gene more closely using other datasets. The boxplot, density distribution plot, and violin plot for PTBP1 are shown for both severe asthma and healthy groups in Fig. 6. Also, the PTBP1 gene expression of GSE69683, GSE76262, and GSE110551 are presented in Fig. 6a–c, respectively. The same mentioned meaningful difference between the two categories is visible in the PTBP1 expression plots for the GSE69683 dataset. A comparison of medians, interquartile ranges, and whiskers for GSE76262 reveals an insignificant difference. This is predictable due to the sampling source of this dataset, sputum, which is considered noisy, because of the presence of many other cells. Hence, GSE69683 plots are more precise, since their data comes from blood samples, and the PTBP1 is more present in the blood.

The GSE110551 is another dataset available for severe asthma studies. It contains BMI scores as well, allowing us to study possible relationships between obesity and severe asthma at the gene level. We investigated the model’s performance on this dataset in two aspects. At first, groups of healthy (non-asthmatic and non-obese) and severe asthma (asthmatic and non-obese) were observed. The larger box length and interquartile range for the healthy group indicate more spread-out data points. A smaller interquartile range for the severe asthmatic group suggests that data is more centered and less dispersed. By paying closer attention to the whiskers, a clear spread and difference in point distributions of asthmatic and healthy group categories can be noticed.

**Figure 7:**
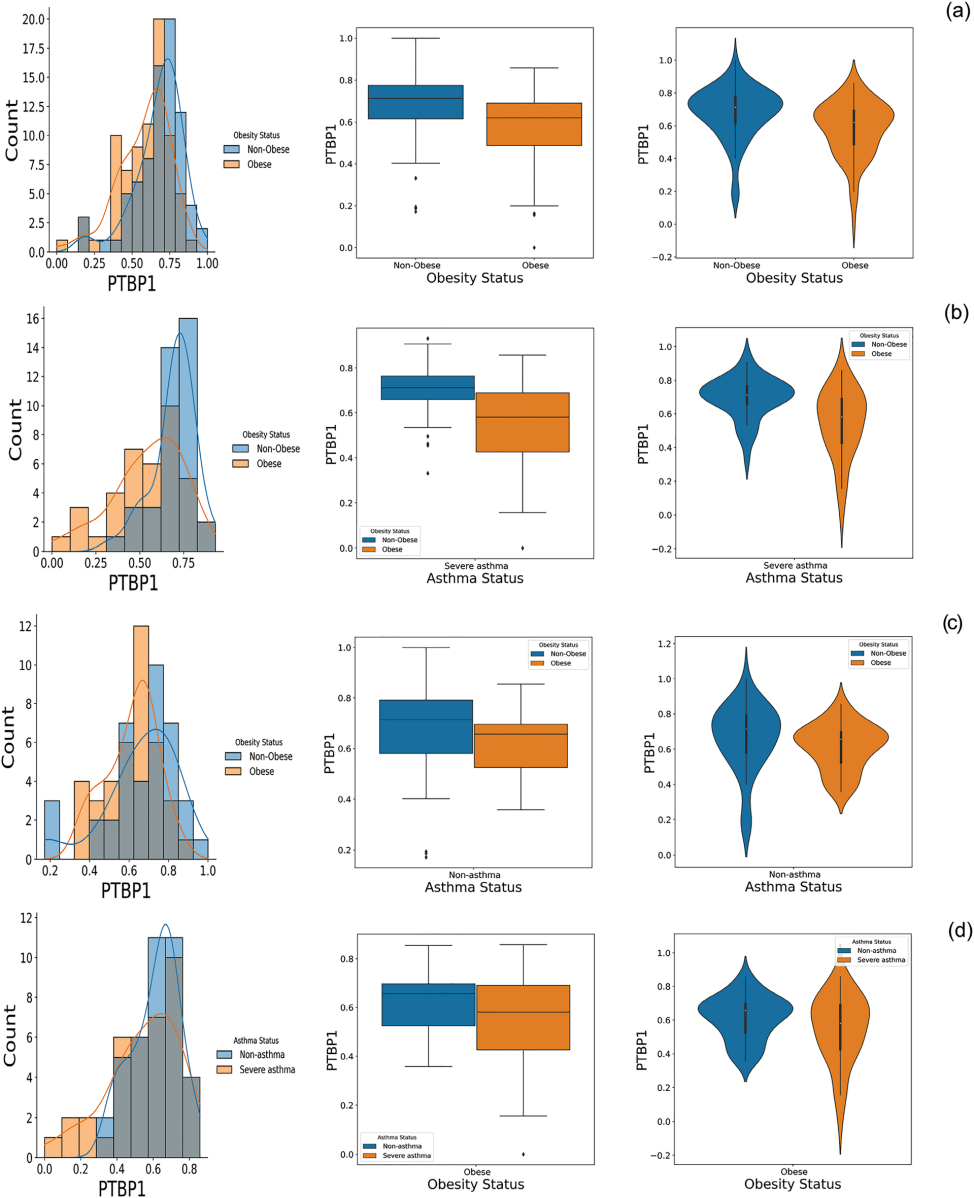
The comparison of the PTBP1 expression based on asthma and obesity status in the GSE110551 dataset. (**a**) Probability Density Function plot, Box plot, and Violin plot of PTBP1 expression for obese and non-obese groups. (**b**) Probability Density Function plot, Box plot, and Violin plot of PTBP1 expression for obese and non-obese groups with severe asthma. (**c**) Probability Density Function plot, Box plot, and Violin plot of PTBP1 expression for obese and non-obese groups with non-asthma. (**d**) Probability Density Function plot, Box plot, and Violin plot of PTBP1 expression for severe asthma and non-asthma groups with obesity.

Afterward, we studied the PTBP1 expressions in severe asthmatic and non-asthmatic groups, by considering obesity status; the corresponding probability density function plot, box plot, and violin plot reveal that obesity as internal stress, affects PTBP1 expression level in samples (Fig. 7a,b). In Fig. 7c and d, the PTBP1 expression box plot displays the poor difference between medians, interquartile ranges, and whiskers.

**Figure 8:**
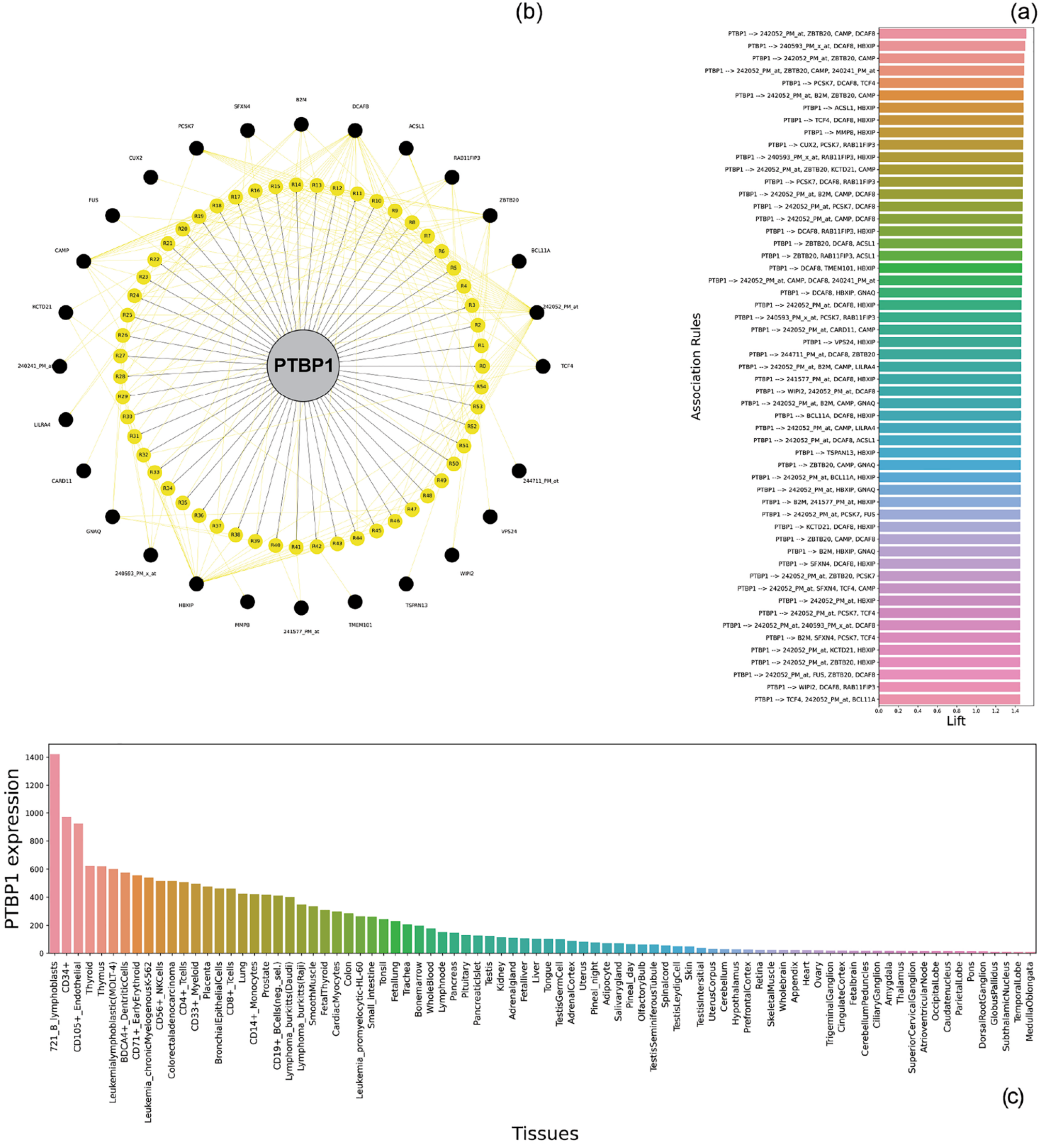
The relationship and specification of the PTBP1 association rules in the GSE69683 dataset. (**a**) Bar plot of lift values of PTBP1 association rules, generated by Apriori algorithm with minimum support 0.28 and minimum lift 1.42. (**b**) Graph network of PTBP1 association rules, in which PTBP1, rules, probes/genes are presented, by gray, yellow, and black colors, respectively. (**c**) Bar plot of PTBP1 expression in various tissues of the human body^37^.

According to the insights extracted from data and the profound effect of the PTBP1 in severe asthma and its susceptibility to various stressors such as obesity and hypoxia, we selected 55 association rules in which the PTBP1 appeared as the antecedent part. The PTBP1 association rules based on lift measure are displayed in Fig. 8a (having minimum threshold values of 1.42). Additionally, a graph network for the PTBP1 is shown in Fig. 8b, which can help improve understanding of the PTBP1 gene interactions in severe asthma disease. More in-depth coverage of these findings is available in the discussion section of the study. Also, the Bar plot of PTBP1 expression in various tissues of the human body was illustrated in Fig. 8c^37^.

## Discussion

In the present work, 100 candidate mRNAs were identified using the ANOVA approach, considering their strong rules and correlation with asthma severity. Utilizing association rule mining, 16 candidate mRNAs were found that have more than 2 repetitions in asthma association rules. Through in-depth functional analysis of asthma candidate genes in literature and research surveys, the PTBP1, RAB11FIB3, APH1A, and MYD88 demonstrated promise as important factors in the development and pathogenesis of severe asthma, potentially involved in the airway hyperresponsiveness, inflammation, and remodeling.

**Figure 9:**
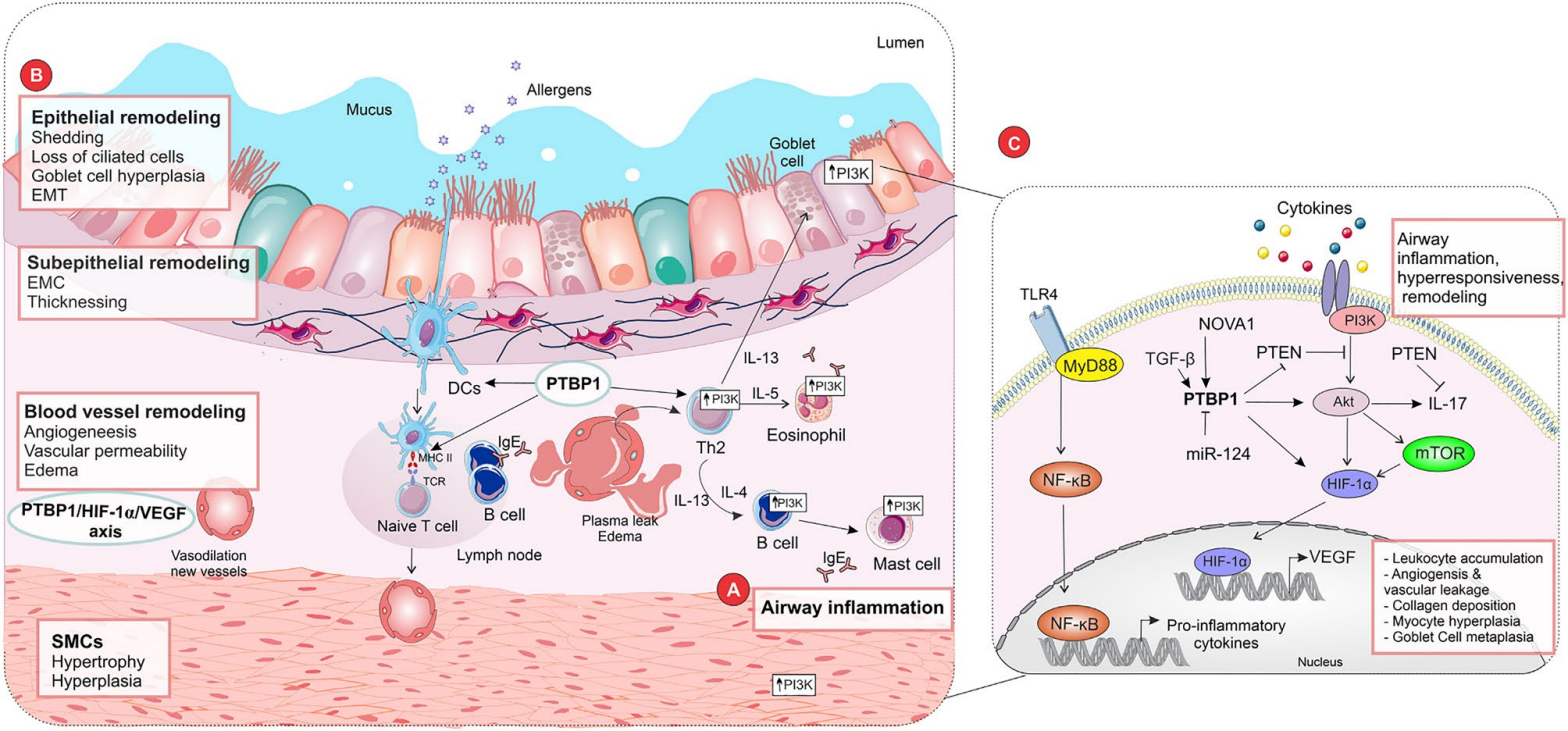
Hallmarks of severe asthma and the possible role of the identified mRNAs by artificial intelligence methods. (**a**) Airway hyperresponsiveness and inflammation and (**b**) airway remodeling (epithelial layer alterations, thickness of airway sub epithelium and smooth muscle (ASM) along with bronchial neo angiogenesis) are involved in the pathogenesis of severe asthma. (**c**) The PTBP1 is an RNA-binding protein that is implicated in several biological pathways, particularly in the activation and regulation of the immune system. The PTBP1, as an alternative splicing factor, could affect different aspects of asthmatic immune responses, notably by increasing MCH II class and disturbing T cell hemostasis that can increase the intensity of the condition from moderate asthma to severe asthma. Moreover, the PTBP1 plays a key role in airway remodeling by inhibiting PTEN and stimulating AKT phosphorylation that can promote PI3K/PTEN/AKT/mTOR/HIF-1/VEGF signaling pathway. TGF-β, NOVA1, and miR-124 can regulate the PTBP1. The MYD88 plays a crucial role in the immune response and functions as a remarkable member of TLR signaling pathways and IL-1, controlling the activation of several pro-inflammatory genes. The TLR4/MyD88/NF-κB signaling pathway has a crucial role in the pathogenesis of asthma. *DCs* Dendritic cells, *HIF-1α* hypoxia-inducible factor-1 alpha, *NF-κB* nuclear factor-kappa b, *NOVA1* neuro-oncological ventral antigen 1, *PI3K* phosphoinositide 3-kinase, *PTBP1* polypyrimidine tract-binding protein, *PTEN* phosphatase and tensin homolog, *SMCs* smooth muscle cells, *TCR* T-cell receptor, *TGF-β* transforming growth factor-beta, *TLR4* Toll-like receptor 4, *VEGF* vascular endothelial growth factor, *mTOR* mammalian target of rapamycin.

Based on **o**ur results, the PTBP1 [polypyrimidine tract-binding protein 1; also known as hnRNP1 (heterogeneous nuclear ribonuclear protein I)] ranked as the 1^st^ important gene in mediating severe asthma (Fig. 9). PTBP1 is an RNA-binding protein with versatile molecular functions related to RNA splicing and metabolism^38^. The PTBP1 is the main known repressive regulator of posttranscriptional gene expression that regulates mRNA stability, splicing, localization, and translation^39^. It involves the processing of mRNAs, affecting the cleavage of 3′-end and alternative polyadenylation^38,40^. Moreover, PTBP1 regulates the expression of several transcripts in different cells via its interactions with microRNAs (miRs). miR-326 by targeting the PTBP1 stimulates autophagy to lessen pulmonary fibrosis^41^. The PTBP1 also regulates cellular migration, proliferation, and apoptosis through different pathways^42^. Additionally, it regulates the alternative splicing of its downstream target genes involved in cell growth and DNA damage^43^.

The imbalance of Th17/ regulatory T cells (Treg) and Th1/Th2 is a key factor in the pathogenesis of asthma^15^. Since alternative splicing of RNAs has vital roles during the maturation and activation of immune cells, abnormal splicing due to PTBP1 dysregulation can trigger autoimmune diseases. Researchers have reported that a specific deficiency of the PTBP1 in dendritic cells (DCs) can elevate the expression of MHC II and disturb T-cell homeostasis without impacting the development of the DC. However, it could increase the populations of memory and activated T cells. In an asthma mice model, deletion of *Ptbp*1 could elevate the immune response by immune cell recruitment, mainly, eosinophils into the lungs, inducing lung damage^44^. Evidence also indicates that PTBP1 is involved in the activation of T-cells by targeting different molecules and mechanisms. This protein is needed for the optimal proliferation, expansion, activation, and survival of T cells. Moreover, optimal expression of T cell activation markers (IFN-γ, TNF-α, IL-2, CD25, CD69, and CD40 ligand (CD40L) is dependent on the PTBP1. It functions as a critical regulator of CD4 T-cell activation that controls the expression of IL-2 and CD40L via the activation of the nuclear factor-κB (NF-κB) and phospholipase Cγ1 (PLCγ1) pathways. Downregulation of the PTBP1 leads to a reduction of these signaling pathways in T cells, preceding alterations in cell division and cytokine expression^45^. Moreover, the PTBP1 regulates the expression of a regulator of the T-cell receptor (TCR), CD5, via different polyadenylation^46^. The PTBP1 participates in the regulation of the CD46 alternative splicing as well^47^. The PTBP1 is also upregulated in B lymphocytes and has important roles in B cell receptor-mediated antibody production^48^ and B-cell selection in germinal centers^49^. Given the roles of PTBP1 in the regulation of different processes of immune cells, its impairment may lead to immune dysregulation in asthma.

Beyond dysregulated immunity and inflammation, structural alterations of the bronchial wall (airway remodeling) are involved in the pathogenesis of asthma, starting from the initial stages of the asthmatic natural history^50^. Epithelial alterations, the thickness of airway subepithelium, and smooth muscle (ASM) along with bronchial neoangiogenesis are hallmarks of asthmatic bronchial remodeling. Epithelial injury/repair cycles are significant signs in asthma airway remodeling, which are followed by metaplasia/hyperplasia of mucus-producing goblet cells, sub-epithelial fibrosis development, epithelial-mesenchymal transition (EMT), and basal membrane thickness. In addition to the abovementioned damage^51^. The Th2 cytokines, particularly IL-13, are triggers of mucus production and goblet cell hyperplasia. Among cytokines, TGF-β is the most potent inducer of the EMT and fibroblast to myofibroblast transition (FMT) along with subepithelial and ASM remodeling, wherein downregulates PTEN (phosphatase and tensin homolog), a phosphatase^52,53^.

Vascular endothelial growth factor (VEGF) has the most significant pathogenic role in asthmatic vascular permeability, remodeling, and angiogenesis. The VEGF is produced by inflammatory (mast cells, macrophages, and eosinophils) and endothelial cells in response to different stimuli, especially hypoxia-inducible factor-1 alpha (HIF-1α)^54^. Activation of the HIF-1α under hypoxia increases inflammation and airway hyperresponsiveness via CD8^+^ type 2 cytotoxic T cells^55^. In pulmonary endothelial cells, an elevated expression of HIF-1α is associated with alterations in nitric oxide and cellular metabolism that are the hallmarks of pulmonary hypertension^56^. Moreover, in the inflammatory leukocytes, HIF-1 supports energy metabolism to prevent ATP depletion^57^ by upregulating pyruvate kinase muscle 2 (PKM2). By stabilizing HIF-1α mRNA^58^, the PTBP1 plays important roles in PKM splicing, regulating the PKM1/PKM2 ratio, PKM2 generation, and a metabolic switching from oxidative phosphorylation to glycolysis^59^, an early asthmatic event^60,61^. A decreased expression of miR-124 in endothelial cells of the pulmonary artery deregulates the splicing of PTBP1 and its target (PKM2), leading to hyperproliferation of endothelial cells^62^. Moreover, in the pulmonary hypertensive vessel, the inflammatory, proliferative, and metabolic states of fibroblasts are regulated by miR-124, PTBP1, and PKM signaling^62^. It is also reported that PKM2 induces the expression of proinflammatory factors and the glycolysis-inactive form of PKM2 has an important function in the pathogenesis of asthma^61^.

At the molecular level, different signaling pathways are involved in the pathogenesis of asthma. Protein kinase C-delta (PKC-δ) induces proinflammatory cytokine production via the NF-κB pathway, indicating its regulatory role in airway inflammation^63^. The PKC-δ is a positive upstream controller of phosphoinositide 3-kinase (PI3K)/Akt/ mammalian target of rapamycin (mTOR)/HIF-1α/VEGF pathway in asthma^64^. Evidence indicates that PI3K has essential roles in different aspects of asthma through HIF-1α-mediated VEGF expression^65,66^. PTEN has also an impact on asthma^67^ by controlling cytokine signaling and different signaling pathways^68^; the PI3k/Akt pathway is mainly inhibited by PTEN^69^. A recent report indicated that overexpression of the PTBP1 could decrease the PTEN expression and elevate the phosphorylation level of Akt significantly, inducing proliferation and migration of asthmatic airway ASM cells^70^. The PTBP1 is itself positively regulated by neuro-oncological ventral antigen 1 (NOVA1), an RNA-binding protein^70^. It is worth noting that the mammalian target of rapamycin (mTOR) signaling is another necessary factor for the initiation of HIF-1α activity and VEGF expression. The PTBP1 also regulates cellular migration, proliferation, and apoptosis through different molecules and pathways^42^. Additionally, it regulates the alternative splicing of its downstream target genes involved in cell growth and DNA damage^43^.

The PTBP1 is involved in several mechanisms and pathways including motility and cell structure, localization and protein targeting, protein modification and metabolism, cell cycle, immunity, muscle contraction, and so on; it might be upregulated by TGF-β1 through C-MYC in Keloids, a connection that can be a possible pathogenic mechanism for fibrotic disease. Moreover, the response of fibroblasts to the TGF-β induces the PTBP1 activation in Keloid^71^. It was demonstrated that miR-124–mediated downregulation of cell proliferation was due to its effects on the PTBP1; this can exert exquisite regulation of many downstream molecules that are important in cell proliferation, such as cell cycle-related genes FOXO3, Notch1, PTEN, p27Kip1 and p21Cip1^62,72–74^.

Evidence indicates a cause-effect and organ-organ interaction between the lung and adipose tissue. Obese subjects have an expanded chance of asthma and stout asthmatics have serious exacerbations, diminished reaction to a few asthmatic solutions, and diminished quality of life^75,76^. The major alterations linked with obesity include activation of the immune system and a positive energy balance^77^. This bidirectional control between inflammatory and metabolic pathways stimulates a movement from obesity to asthma severity.

To date, there is a lack of reports linking the PTBP1 with asthma and obesity. However, there is some evidence to indicate how the impairment of PTBP1 may lead to obesity. A long non-coding RNA (H19) has an important role in the metabolism of lipids, where its upregulation can improve insulin sensitivity and protect against obesity^78^. The PTBP1 can interact with H19 to reprogram lipid homeostasis in the liver^79^. On the other hand, the PTBP1 is needed for cleavage, activation, and translocation of the SREBP1 (sterol-regulatory element binding proteins). The SREBP1 is a transcription factor that regulates genes involved in the glycolysis and de novo lipogenesis pathways, leading to hepatic accumulation of lipid and insulin resistance^80^. In obese patients, an elevated level of SREBP1 is associated with insulin resistance and hepatic steatosis^81^. Interaction of the PTBP1 with H19 blocks its function, inhibiting the cleavage of the SREBP1 precursor. However, nuclear translocation of the SREBP1 in the absence of H19, stimulates the transcription of lipid-related genes, resulting in the accumulation of lipid^82^.

PTBP1 can significantly affect airway inflammation and remodeling in asthma by modulating the PI3K/PTEN/AKT/mTOR/HIF-1/VEGF signaling pathways. The chronic asthma phenomena can be a result of cytokines/chemokines, especially TGF-β’s effect on the PTBP1 expression pattern and subsequently, its impact on key signaling molecules. Hallmarks of severe asthma and the possible role of the identified mRNAs by artificial intelligence methods are displayed in Fig. 9.

Our study has some limitations. First of all, we did not evaluate the expression of the identified genes in clinical samples. Moreover, the molecular mechanism of the identified RNAs was not evaluated in the asthma models. Further experimental and clinical studies are needed to be performed to achieve these goals. It should be also noted that due to their well-known accuracy, gene network analysis and Machine learning methods have been applied widely for discovering novel diagnostic, prognostic, and therapeutic targets in the realm of asthma. Moreover, plenty of genes have been identified to have key roles in the pathogenesis of asthma^17,83–86^. Some other genes such as thyroid peroxidase and superoxide dismutase 2 were identified to play important roles in asthma^87,88^. However, it is challenging to locate the exact genes complicated in a complex asthma disease owing to the nature of a gene’s multiple functions and heterogeneous mechanisms of the severe disease^85,89^.

## Conclusion and perspective

We analyzed the blood transcriptome profiles of severe asthma patients and compared them with healthy controls, using artificial intelligence approaches (e.g., ANOVA, deep learning, and association rule mining). Our findings determine the PTBP1 as a main candidate gene and the most frequent item among asthma association rules. Given the discussed pluripotent roles of the PTBP1, it is reasonable to speculate that PTBP1 may regulate the immune cells, proinflammatory responses, hypoxia-related cellular metabolism, and airway remodeling in asthma. Also, the PTBP1 affects the basic mechanisms of pre-mRNA splicing including spliceosome assembly, miRNA synthesis and maturation, and the expression, activity, and intracellular localization of splicing factors that can trigger severe asthma. PTBP1 may also establish a strong bridge between asthma and obesity. The notion of moving from traditional treatments toward more novel therapeutic strategies such as RNA-based targeted therapy can open a new horizon in medicine to overcome asthma and obesity disorders where there are no boundaries.

### Supplementary Information

Supplementary Information.

## Data Availability

The data obtained from the artificial intelligence approaches will be available from the corresponding authors upon request.
