# Supplementary material for: Unraveling the link between PTBP1 and severe asthma through machine learning and association rule mining method
Source: Sci Rep. 2023 Sep 16;13:15399. doi: 10.1038/s41598-023-42581-5 (PMC10505163; doi:10.1038/s41598-023-42581-5)
Supplement: Supplementary file 1 — Supplementary Information. [file 41598_2023_42581_MOESM1_ESM.pdf]

Unraveling the Link between PTBP1 and Severe Asthma through Machine Learning and Association Rule Mining Method

Saeed Pirmoradi, Ph.D. [1†], Seyed Mahdi Hosseiniyan Khatibi, Ph.D. [2, 3 †], Sepideh Zununi Vahed, Ph.D. [2 †], Hamed Homaei Rad, Msc[3†], Amir Mahdi Khamaneh, Ph.D.[4], Zahra Akbarpour, Msc[3], Ensiyeh Seyedrezazadeh, Ph.D.[5], Mohammad Teshnehlab, Ph.D.[6], Kenneth R. Chapman, MD [7] *, Khalil Ansarin, MD [3] *

[1] Clinical Research Development Unit of Tabriz Valiasr Hospital, Tabriz University of Medical Sciences, Tabriz, Iran

[2] Kidney Research Center, Tabriz University of Medical Sciences, Tabriz, Iran.

[3] Rahat Breath and Sleep Research Center, Tabriz University of Medical Science, Tabriz, Iran.

[4] Faculty of Advanced Medical Sciences, Tabriz University of Medical Sciences, Tabriz, Iran.

[5] Tuberculosis and Lung Disease Research Center, Tabriz University of Medical Sciences, Tabriz, Iran.

[6] Department of Electric and Computer Engineering, K.N. Toosi University of Technology, Tehran, Iran.

[7] Division of Respiratory Medicine, Department of Medicine, University of Toronto, Toronto, Ontario, Canada.

***Corresponding authors:**
Khalil Ansarin
Rahat Breath and Sleep Research Center, Tabriz University of Medical Science, Tabriz, Iran.
Email: dr.ansarin@gmail.com

Kenneth R. Chapman
Division of Respiratory Medicine, Department of Medicine, University of Toronto, Toronto, Ontario, Canada.
Email: ken.chapman.airways@gmail.com

† These authors contributed equally and should be considered as co-first authors.

Supplementary Method

1. ANOVA

ANOVA is a simple and powerful method to compare the mean value of multiple groups (classes) in a dataset. It highlights any significant difference between the mean values of groups [1]. This method has many advantages including: being robust in point view violations of its assumptions, being more intuitive to analyze the interaction of two features, being effective even in datasets with imbalanced number of samples in target classes, and also being easy to generalize to more than two groups without increasing the Type 1 error. ANOVA is called F-statistic in statistics literature, and is calculated by Eq. 1 [2].

$$F_{value} = \frac{BMS}{WMS} \qquad (1)$$

In Eq. 1, BMS and WMS are between mean squares and within mean squares, respectively. BMS and WMS are calculated by Eqs. 2 and 3.

$$BMS = \frac{BSS}{df_B} = \frac{\sum_{i=1}^{C} n_i(\bar{x}_i - \bar{x})^2}{df_B} \qquad (2)$$

$$WMS = \frac{WSS}{df_W} = \frac{\sum_{i=1}^{C} (n_i - 1)\sigma_i{}^2}{df_W} \qquad (3)$$

BSS and WSS are between sum squares and within sum squares, respectively. Where $\bar{x}$ = mean value of total samples, $\bar{x}_i$ = mean value of ith class, σ_i = standard deviation of ith class, n_i = sample number of ith class. df_B = C-1 and df_W = N-C represent degree of freedom, in which N = number of total sample and C = Number of classes.

ANOVA assigns calculated F-values for all features and performs the ranking process accordingly. Features with high F-values are significant, since they represent better differentiation capabilities between classes.

2. Self-Organizing Deep Auto-Encoder

Auto-encoder is a specific type of artificial neural network mainly used for dimensionality reduction purposes. In recent years, auto-encoders have proven to be successful in bioinformatics applications, as well as identifying and classifying biological patterns in genomics data. The auto-encoder structure contains two layers of encoding and decoding, as shown in Figure 1. The output of the encoding layer generates the decreased or increased representation of the input vector. The

decoding layer on the hand, reconstructs the initial input vector, based on the output vector of encoding layer.

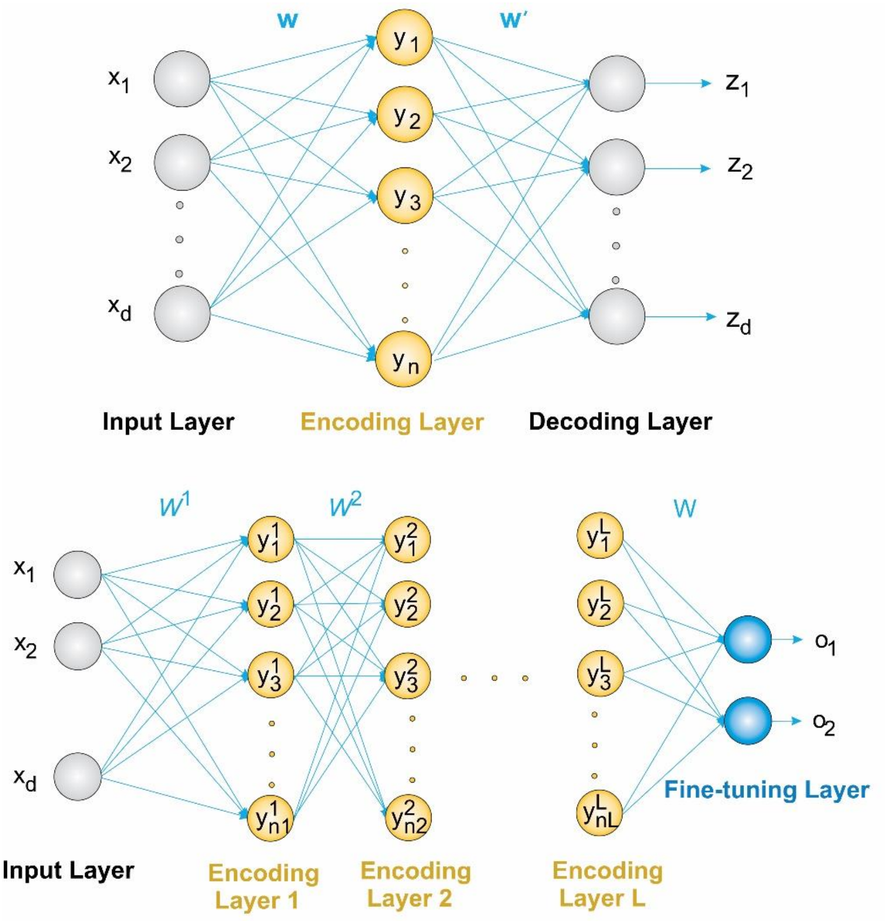

Figure 1. The Deep Model architecture. a) Self-Organizing Auto-Encoder (SOAE) is defined as a new type of AE, that its training algorithm can automatically determine AE structure according to input data. The algorithm can estimate the number of layers and nodes in SOAE. b) The deep auto-encoder model is constructed by merging two parts, including representation layers and fine-tuning layer. Representation layers are built by stacking auto-encoders as building blocks. In the fine-tuning layer, the deep model utilizes classifier models.

The encoding layer maps an input vector $X \in [0\ 1]^d$ to an encoding vector $Y \in\ [0\ 1]^n$ in the hidden layer (or representation layer) using the $f(x)$ function defined by:

$$Y = f_\theta(X) = S_f(WX + b) \qquad (4)$$

S_f is a non-linear activation function and the deterministic function $Y = f_\theta(X)$ is parameterized by $\theta = \{W, b\}$, as is a weight matrix in which b represents a bias vector.

The decoding layer maps back the representation vector $Y \in\ [0\ 1]^n$ to a reconstruction vector $Z \in [0\ 1]^d$, using the $g_\theta(X)$ function shown by Eq. 5.

$$Z = g_{\theta}(Y) = S_g(W'Y + b') \qquad (5)$$

S_g is a linear activation function and the deterministic mapping $Z = g_{\theta}(Y)$ is parameterized by $\theta' = \{W', b'\}$. The weight matrix W' can be constrained by $W' = W^T$, that contains tied weights of AE.

The parameters of the AE are optimized to minimize the loss function L, as shown in Eqs. (6) and (7).

$$\theta.\theta' = \underset{\theta.\theta'}{\operatorname{argmin}} \frac{1}{N} \sum_{i=1}^{N} L(x^{(i)}.z^{(i)}) \qquad (6)$$

$$\theta.\theta' = \underset{\theta.\theta'}{\operatorname{argmin}} \frac{1}{n} \sum_{i=1}^{n} L(x^{(i)}.g_{\theta'}(f_{\theta}(x^{(i)}))) \qquad (7)$$

L can be defined as the traditional squared norm error $L(x.z) = ||x - z||^2$. Another choice for loss function is cross-entropy that uses sigmoid function as S_g and its input vector is in [0 1] range. The cross-entropy loss function is shown in Eq. (8).

$$L(x.z) = -\sum_{i=1}^{N} x^{(i)} \log\left(z^{(i)}\right) + (1 - x^{(i)}) \log\left(1 - z^{(i)}\right) \qquad (8)$$

The deep auto-encoder model is formed by merging two parts, representation layers and fine-tuning layer. Representation layers are built by stacking auto-encoders as building blocks. Deep model utilizes classifier models, such as neural networks, support vector machines, etc. for the fine-tuning layer, and the classification is performed on representation input data obtained from representation layers. The deep auto-encoder model is shown in Figure 2b where x_i is the ith feature in input vector X, y_i^j is the output of ith neuron in j^{th} layer, and O_i is the output of fine-tuning layer. Auto-encoder models are pretty powerful high-capacity artificial neural networks that can only be controlled either by composition of their structures, or applying regularization methods. A SOAE is defined as a new type of auto-encoders that is capable of determining AE structure according to input data [3]. Estimating the number of layers and nodes are done algorithmically in a SOAE; therefore, manual testing of various network architectures is no longer needed during model building process. This helps minimize both time and computational demands. SOAEs can also be beneficial in diminishing over-fitting problem, by avoiding over-complex and inefficient structures, and building up the just-right size of layers and nodes based on complexity of the task. This study takes advantage of the deep model based on a SOAE in order to tackle classification.

3. Association Rule Mining

Association rule mining is traditionally applied to market basket data and is defined as an analysis framework. It can help producers or retailers to identify new opportunities for selling their products to customers, by digging up data to obtain knowledge from association analysis. AIS was the first algorithm introduced for association rule mining analysis [4], but failed to meet expectations due to excessive generation of unnecessary itemsets. Subsequently, the Apriori algorithm was proposed by R. Agrawal et al. [5], which also uses the frequent itemsets concept, but with pruning techniques to avoid measure bound itemsets. Apriori is probably the most used association rule mining algorithm to date; however, several improved algorithms are proposed as well, such as the Apriori-Hybrid algorithm [6], fuzzy association rule algorithm [7] and FP-Growth algorithm [8, 9].

An association rule is defined in $X \rightarrow Y$ form, where $X.Y \subseteq I$, $X \cap Y = \varphi$ and I is the set of all items. The left side (X) and the right side (Y), are called "antecedent" and "consequent" in an association rule, respectively. The $X \rightarrow Y$ rule means that if X occurs, Y occurs very likely.

Association rule mining can help identify the biological relationship between genes, or environmental stimuli and gene expression levels, or even biological information about genes and gene expression levels [10]. For example, $gene_A \rightarrow gene_B.gene_C$ as an association rule can be interpreted in this way: if $gene_A$ is over-expressed, $gene_B$ and $gene_C$ are very likely over-expressed.

3.1. Frequent Pattern Mining (FPM)

Frequent itemsets are elements with high level of importance in data mining literature. They can contain significant patterns hidden in data, including association rules. During association rule mining process, FPM techniques automatically extract frequent itemsets from the dataset and use them to generate association rules. The problem with FPM techniques is that they generate enormous frequent itemsets, and it is a costly task eventually. The search space is exactly equal to $2^{|I|}$ different itemsets. Therefore, discovering all frequent patterns becomes a challenging burden. Let I = $\{i_1, i_2, \ldots, i_d\}$ and T = $\{t_1, t_2, \ldots, t_N\}$ be the set of all items and the set of all instances/ transactions, respectively. Each instance contains the subset of items from the "I" set. In association analysis, an itemset with "k" items is called k-itemset, and each itemset has an important property, called *support count*. The support count of an itemset means the number of instances that contain that itemset, as shown in Eq. 9 in mathematical notation.

$$Support_{count} = |\{t_i | X \subseteq t_i \,.\, t_i \in T\}| \qquad (9)$$

An itemset S, $S \subseteq I$, is called a frequent itemset: if S is present in at least a fraction of all transactions in T; a user-defined threshold, namely "minimum support count" specifies that fraction.

3.2. Support, Confidence and Lift of a Rule

An association rule is represented by $X \rightarrow Y$, in which both X and Y are itemsets. The strength of an association rule is measured using *support* and *confidence* benchmarks. Support indicates "how a rule is applicable" and confidence marks "the appearance frequency of Y items in instances that contain X". The formulas for these metrics are shown in Eqs. 10 and 11.

$$Support(X \rightarrow Y) = \frac{Support_{Count}(X \cup Y)}{N} \qquad (10)$$

$$Confidence(X \rightarrow Y) = \frac{Support_{Count}(X \cup Y)}{Support_{Count}(X)} \qquad (11)$$

Lift is a correlation measure that can be used in association analysis and is calculated by Eq. 12 for X and Y itemsets. The $X \rightarrow Y$ association rule with Lift(X, Y) has three states:

A) Less than 1 ($Lift < 1$): the occurrence of X is correlated negatively with the happening of Y.

B) More than 1 ($Lift > 1$): the occurrence of X is correlated positively with the happening of Y.

C) Equal to 1 ($Lift = 1$): the occurrence of X is not correlated with the happening of Y (they are independent of each other).

$$Lift(X.Y) = \frac{P(X \cup Y)}{P(X)P(Y)} \qquad (12)$$

3.3. Apriori Algorithm

Apriori algorithm is the first association rule mining algorithm that applies the support metric for pruning candidate itemsets. This characteristic controls the exponential growth of candidate itemsets. The input for the Apriori algorithm is a N×M data matrix. Rows and columns of the matrix are corresponded with instances (or samples) and gene expression levels (or features). Each feature in the gene expression data matrix is converted to discrete values in phase I of the process. This transformation needs to be applied, since association rule mining algorithms can only work with binary values. During the discretization process, the values for each feature are categorized into some discrete bins. In order to do so, a basic unsupervised strategy would be to use equal width partitioning, throughout which continuous features are divided into k bins with an equal size (k is a user-defined parameter).

In phase II, the Apriori algorithm iteratively discovers all frequent itemsets that satisfy the minimum user-defined support threshold value. FPM is the most costly step in terms of computing and storage space. The pseudocode for generating frequent itemsets is presented in Table 1.

The phase III of the Apriori algorithm involves construction of association rules based on frequent itemsets; again, these rules should satisfy a user-defined minimum threshold benchmark, such as “confidence” or “lift”. Within this phase, any frequent itemset is separated into two itemsets: X and Y; later the related rule is constructed in the form of $X \rightarrow Y$. Only the rules with a confidence (or lift) value higher than threshold are kept. Also, the threshold can be used to regulate and reduce the number of association rules; since any frequent itemset can generate $2^k - 2$ association rules (k is equal to number of elements frequent itemset), the number of total rules could increase in an exponential manner if not regulated. The pseudocode for construction of association rules is shown in Table 2.

Finally, the last phase includes filtering generated rules according to the research criteria and shift the focus onto studying significance of mined associations.

Table 1. Pseudocode of frequent itemsets generation step in Apriori algorithm [11]

**Algorithm:** Frequent itemsets generation in Apriori algorithm
**Method:**
k=1;
$F_k = \{i \mid i \in I \wedge Support_{Count}(i) \geq N \times minsup\}$; // Find all frequent 1-itemsets
**Repeat**
k = k+1;
C_k = candidates generated from F_{k-1};
**For** each each instance $t \in T$ do
$C_t = subset(C_k.t)$; // Identify all candidates that belong to t
**For** each candidate itemset $c \in C_t$ **do**
$Support_{Count}(c) = Support_{Count}(c) + 1$; // Increment support count
**End for**
**End for**
$F_k = \{c \mid c \in C_k \wedge Support_{Count}(c) \geq N \times minsup\}$; // Extract the frequent k-itemsets
**Until** $F_k = \phi$;
Result = $\cup F_k$;

Table 2. Pseudocode of rules generation step in Apriori algorithm [11]

**Algorithm:** Rules generation in Apriori algorithm
**Method:**<br>1- **For** each frequent itemset k-itemset F_k, k≥2 **do**<br>2- $H_1 = \{i \mid i \in F_k\}$; // 1-item consequents of the rule.<br>3- call ap-genrules(F_k, H_1);<br>4- **End for**<br><br>Procedure ap-genrules(F_k, H_m)<br>1- $k = \|F_k\|$; // size of frequent itemset<br>2- $m = \|H_m\|$; // size of rule consequent<br>3- **if** k>m+1 **then**<br>4- $H_m = m + 1 - item\ consequents\ generated\ from\ H_m$;<br>5- **For** each $h_{m+1} \in H_{m+1}$ do<br>6- $Confidance = \frac{Support(F_k)}{Support(F_k - H_{m+1})}$;<br>7- **if** Confidence ≥ min_confidence **then**<br>8- output: the rule (F_k – h_{m+1}) → h_{m+1};<br>9- **Else**<br>10- delete h_{m+1} from H_{m+1};<br>11- **End if**<br>12- **End for**<br>13- call ap-genrules(F_k, H_{m+1})<br>14- **End if**

References

[1] T. K. Kim, "Understanding one-way ANOVA using conceptual figures," *Korean journal of anesthesiology*, vol. 70, no. 1, p. 22, 2017.

[2] H.-Y. Kim, "Analysis of variance (ANOVA) comparing means of more than two groups," *Restorative dentistry & endodontics*, vol. 39, no. 1, p. 74, 2014.

[3] S. Pirmoradi, M. Teshnehlab, N. Zarghami, and A. Sharifi, "A Self-organizing Deep Auto-Encoder approach for Classification of Complex Diseases using SNP Genomics Data," *Applied Soft Computing*, p. 106718, 2020/09/12/ 2020, doi: https://doi.org/10.1016/j.asoc.2020.106718.

[4] R. Agrawal, T. Imieliński, and A. Swami, "Mining association rules between sets of items in large databases," in *Proceedings of the 1993 ACM SIGMOD international conference on Management of data*, 1993, pp. 207-216.

[5] R. Agrawal and R. Srikant, "Fast algorithms for mining association rules," in *Proc. 20th int. conf. very large data bases, VLDB*, 1994, vol. 1215: Citeseer, pp. 487-499.

[6] R. Agrawal, H. Mannila, R. Srikant, H. Toivonen, and A. I. Verkamo, "Fast discovery of association rules," *Advances in knowledge discovery and data mining*, vol. 12, no. 1, pp. 307-328, 1996.

[7] C. M. Kuok, A. Fu, and M. H. Wong, "Mining fuzzy association rules in databases," *ACM Sigmod Record*, vol. 27, no. 1, pp. 41-46, 1998.
[8] J. Han, J. Pei, Y. Yin, and R. Mao, "Mining frequent patterns without candidate generation: A frequent-pattern tree approach," *Data mining and knowledge discovery*, vol. 8, no. 1, pp. 53-87, 2004.
[9] J. Han, J. Pei, and Y. Yin, "Mining frequent patterns without candidate generation," *ACM sigmod record*, vol. 29, no. 2, pp. 1-12, 2000.
[10] S. Naulaerts *et al.*, "A primer to frequent itemset mining for bioinformatics," *Briefings in Bioinformatics,* vol. 16, no. 2, pp. 216-231, 2013, doi: 10.1093/bib/bbt074 %J Briefings in Bioinformatics.
[11] J. Xie, J. Wu, and Q. Qian, "Feature selection algorithm based on association rules mining method," in *2009 Eighth IEEE/ACIS International Conference on Computer and Information Science*, 2009: IEEE, pp. 357-362.
